# Salivary flow and xerostomia in older patients with type 2 diabetes mellitus

**DOI:** 10.1371/journal.pone.0180891

**Published:** 2017-08-02

**Authors:** Danilo Lopes Ferreira Lima, Sandro Dias Rocha Mendes Carneiro, Fladia Taciana de Sousa Barbosa, Maria Vieira de Lima Saintrain, Jean André Hervé Moizan, Jean Doucet

**Affiliations:** 1 Dental School Undergraduation and Graduation Programs, University of Fortaleza – Unifor, Fortaleza, Brazil; 2 Dental School, University of Fortaleza – Unifor, Fortaleza, Brazil; 3 Dental School Graduation Program, University of Fortaleza – Unifor, Fortaleza, Brazil; 4 Public Health Graduation Program, University of Fortaleza – Unifor, Fortaleza, Brazil; 5 Dental Care Department, Rouen University Hospital, Saint Julien Hospital, Rouen, France; 6 Department of Internal Medicine, Geriatrics and Therapeutics, Saint Julien Hospital, Rouen University Hospital, Rouen, France; Boston University Henry M Goldman School of Dental Medicine, UNITED STATES

## Abstract

**Objectives:**

To assess salivary flow in older patients with type 2 diabetes mellitus (DM2) and its association with xerostomia.

**Methods:**

Cross-sectional clinical study conducted with older patients diagnosed with type 2 diabetes for at least one year receiving treatment at the Integrated Center for Diabetes and Hypertension of Ceará (CIHD) in the city of Fortaleza, Ceará, Northeastern Brazil. Oral clinical examination was carried out to assess the decayed, missing and filled teeth index (DMFT). Perception of the presence of xerostomia/dry mouth was assessed using the Visual Analogue Scale. Stimulated salivary flow was measured and samples were obtained using an extra-soft silicone device.

**Results:**

120 older patients with diabetes (60 insulin-dependent and 60 non-insulin-dependent) aged 65–91 years, with a mean age of 72.26 ± 6.53 years, were assessed. Of these, 111 (92.5%) presented a decrease in salivary flow while 59 (49.2%) reported moderate to severe xerostomia/dry mouth. The DMFT Index presented a mean of 27.53 ± 4.86 teeth.

**Conclusions:**

Reduced salivary flow was found in the group assessed in the present research; however, this finding is not in accordance with the perception of xerostomia/dry mouth reported by the patients.

## Introduction

According to the Global Health Observatory (GHO) of the World Health Organization (WHO), 70 years was the average life expectancy in 2012. In developing countries such as Brazil, there has been a rapid increase in life expectancy in recent decades and data have shown that it has doubled in the past century. It is estimated that 25% of the population will be over the age of 60 years by 2025, compared to 8.6% in 2000 and 4% in 1950 [1].

This rapid and unprecedented increase in human life expectancy is associated with changes in the prevailing patterns of disease and morbidity of conditions that mostly affect the older population. For instance, chronic diseases such as heart disease, dementia, depression, cancer, fall-related injuries and diabetes mellitus (DM) are most likely to affect older individuals [2–4].

Among these pathologies, DM stands out as a common serious age-related disease. It is estimated that 17.4% of Brazilians aged between 60–69 years have diabetes. In the US, over 25% of the population aged ≥65 years have diabetes, while in France the prevalence is estimated to be 14.2% [5,6]. In addition to common chronic complications like heart disease, nephropathy, neuropathy and retinopathy, DM has been associated with oral complications such as periodontal disease (PD), hyposalivation and xerostomia–or dry mouth [7,8].

Saliva is a fluid with many functions such as oral digestion, oral mucosa lubrication, maintenance of the ecological balance in the oral cavity, antimicrobial activity and effective protection of teeth integrity by maintaining the pH in the oral cavity [9]. Hyposalivation affects the quality of life due to the importance of salivary functions. It can be caused by conditions like aging, use of certain drugs, diet, treatment with radiation and certain diseases such as diabetes. Individuals with hyposalivation show a higher prevalence of periodontitis [10].

Given the importance of the oral health status of older people with diabetes, the aim of the present study was to assess salivary flow and its association with xerostomia in older patients with type 2 DM.

## Methods

The study was approved by the Ethics Committee of the Universidade de Fortaleza under Approval No. 788.534. Written consent was provided by the participants (or by their next of kin in case of illiterate people) prior to participation in the research. The participants were aware of the research objectives and anonymity was guaranteed.

This quantitative cross-sectional study was conducted with 120 patients diagnosed with type 2 diabetes for at least one year receiving treatment at the Integrated Center for Diabetes and Hypertension of Ceará (CIHD) in the city of Fortaleza, located in the state of Ceará, Northeastern Brazil. The study included patients, regardless of gender, aged 65 years and older: 60 insulin-dependent patients and 60 non-insulin-dependent patients.

Oral clinical examination was performed to assess dental conditions through the decayed, missing and filled teeth index (DMFT index). The score range 0–32 was used to determine dental conditions [11]. The cause of missing teeth (dental caries, periodontitis, or both) was verified.

Perception of xerostomia was assessed using the Visual Analogue Scale (VAS) validated for patient satisfaction by Brokelman *et al*. [12]. The following guiding question was used: “how dry do you feel your mouth?”. The measurement was performed on a scale from 0 to 10 through which patients indicated the number related to their perception of oral dryness. Values 0–3 indicated none/mild dryness, 4–7 moderate and 8–10 severe.

The salivary flow measurement was performed by having patients chew an extra-soft silicone device. Samples were obtained with the patient sitting comfortably with the head slightly tilted forward. Patients were told to chew the silicone device as it was tied to a 25cm dental floss in order to prevent accidental swallowing. All patients who used dental prostheses were required to wear them during measurement. The saliva produced during a period of 5 minutes was collected and measured in a container graduated at 0.05ml. The volume of salivary flow was calculated in ml/min (millimiter per minute). The patient was instructed not to swallow any amount of saliva that was being produced. Additionally, the patient should keep the body tilted forward in order to avoid saliva swallowing.

Sialometric values were given according to standards established by the Brazilian Association of Halitosis (ABHA): 0.00–0.049 ml/min–asialia; 0.05–0.5 ml/min–severe hyposalivation; 0.51–0.9 ml/min–moderate hyposalivation; 0.91–1.19 ml/min–mild hyposalivation; 1.2–2.5 ml/min–ideal salivation; > 2.5 ml/min–sialorrhea [13].

Data were tabulated and statistical calculations were performed using the Statistical Package for the Social Sciences (SPSS), version 22.0 (SPSS Inc., Chicago, USA). The normality of the distribution of each variable was assessed using the Kolmogorov-Smirnov test. Fisher’s exact test with significance level set at p<0.05 was used to check for associations between categorical variables. The Kruskal-Wallis test with significance level set at p<0.05 was used to check for associations of salivation rating with DMFT and tooth loss. T-test was used to compare DMFT between genders.

## Results

The research included 120 older patients: 38 (31.7%) men and 82 (68.3%) women. Age ranged between 65 and 91 years, with a mean age of 72.26 ± 6.53 years (71.63 ± 5.95 years for men and 72.55 ± 6.80 years for women). There was a prevalence of married individuals (53.3%) and primary education (68.3%).

The time elapsed since diagnosis of DM2 ranged between 1 and 45 years, with a mean of 15.95 ± 9.48 years. Patients who used insulin presented a time of evolution of diabetes of 18.37±9.01 years while those who did not use insulin presented a time of evolution of 13.53±9.4 years (T-test, p = 0.007). No statistical significance was found when genders were compared.

In all, 114 (95%) patients used at least one medication for diabetes control. A total of 113 (94.2%) patients had hypertension and used up to four antihypertensive drugs per day. Of these patients, 48 (40%) used only one medication that could cause dry mouth symptoms, 44 (36.7%) used two or more medications that caused xerostomia, and 28 (23.3%) patients did not use any medication with such effects. No significant statistical association was found between the use of medications that caused xerostomia and salivation status when comparing users and non-users of medications that cause dry mouth.

Table 1 shows that salivary flow rate ranged between 0.05 and 2.1ml/min, with a mean of 0.52±0.42ml/ min. Statistical significance (p = 0.015) was found only between genders and not between insulin-dependent and non-insulin dependent individuals (0.338) (Table 1).

**Table 1:** Mean and standard deviation of salivary flow rate by gender and use of insulin.

Groups	Salivary flow (ml/min) (Mean/SD)	p
**Gender**		
Women	0.47±0.41	0.015
Men	0.63±0.44	
**Use of insulin**		
Yes	0.50±0.43	0.338
No	0.54±0.42	

Source: research data. Mann-Whitney test

In Table 2 it is possible to observe that no statistically significant differences were found between genders (p = 0.381) and use of insulin (p = 0.177) when analyzing the parameters of salivary flow classification. However, a high prevalence of reduced salivary flow–mainly severe hyposalivation–was observed. Additionally, gender (p = 0.947) and use of insulin (p = 0.561) were not significantly associated with xerostomia/dry mouth (Table 2).

**Table 2:** Frequency and distribution of salivary flow classification and perception of xerostomia/dry mouth.

Variable	Women N(%)	Men N(%)	p	Insulin N(%)	Non-insulin N(%)	p
**Salivary flow classification**						
Normal	4 (4.9)	5 (13.2)	0.381	5 (8.3)	4 (6.7)	0.177
Mild Hyposalivation	2 (2.4)	2 (5.3)		3 (5.0)	1 (1.7)	
Moderate Hyposalivation	19 (23.2)	8 (21.1)		8 (13.3)	19 (31.7)	
Severe Hyposalivation	52 (63.4)	23 (60.4)		41 (68.4)	34 (56.7)	
Asialia	5 (6.1)	0 (0)		3 (5.0)	2 (3.2)	
**Perception of xerostomia/dry mouth according to VAS**						
None/mild	41 (50.0)	20 (52.6)	0.947	33 (55.0)	28 (46.7)	0.561
Moderate	37 (45.1)	16 (42.1)		25 (41,7)	28 (46.7)	
Severe	04 (4.9)	02 (5.3)		02 (3.3)	04 (6.6)	

Source: research data. Fisher’s Exact test

### DMFT

The mean DMFT index score of both genders was 27.53±4.86. There was a mean of 0.31±0.9 decayed teeth. Regarding tooth loss, the results showed a mean of 25.28±7.49 missing teeth. There was a mean of 2.06 ± 3.42 filled teeth. The analysis of DMFT (p<0.001) and tooth loss (p<0.001) by gender revealed a statistically significant association with female gender compared to men. Tooth loss occurred due dental caries in 57 (47.5%) patients; periodontitis in 7 (5.8%) patients; and due to both diseases in 56 (46.7%) patients.

Insulin-dependent individuals had a greater number of missing teeth, with a mean of 26.10±6.98 missing components compared to a mean of 24.45±7.94 missing teeth in non-insulin dependent individuals. However, there was no statistically significant difference (Table 3).

**Table 3:** Distribution of the DMFT index and tooth loss by gender and use of insulin.

	DMFT Mean/SD	p	Tooth loss Mean/SD	p
**Gender**				
Men	25.29±5.18	<0.001	20.84±8.12	<0.001
Women	28.57±4.36		27.33±6.23	
**Use of Insulin**				
Yes	28.03±4.85	0.183	26.10±6.98	0.237
**No**	27.03±4.86		24.45±7.94	

Mann-Whitney test

## Discussion

The oral health of patients with diabetes has been widely studied over decades. The studies are primarily focused on the relationship between diabetes and periodontal disease. Initially, the studies have assessed the effects of diabetes on gingivitis and periodontitis; then, the studies sought a bidirectional relationship in which periodontal diseases could hinder glycemic control [14–17, 7], which confirms the importance of dental care for patients with diabetes.

The present study presented a higher prevalence of female individuals, confirming the idea that women are more affected by diabetes than men in Brazil [18]. The higher prevalence of women can be explained by their greater use of health services as well as their greater awareness of the signs and symptoms of the disease and adherence to diagnostic tests [19, 20]. Given the high risk of developing DM2, particularly with aging, the mass screening of asymptomatic individuals should be put into effect in order to predict and prevent such a public health problem in populations. However, effective early detection methods have not been proven yet [21].

It has also been observed that the time elapsed since diagnosis of diabetes was higher among insulin-dependent patients compared to non-insulin-dependent patients, with a mean of 18.37±9.01 years and 13.53± 9.4 years, respectively (p = 0.007). Significant differences were found in a study that sought to identify the same relationship in patients over the age of 30 (9.6±9.2 years for insulin-dependent patients and 6.0±6.1 years for non-insulin-dependent patients, with p = 0.043) [22].

Although findings of Silva et al. [18] revealed that the dental care provided to patients with such diseases is still poor in Brazil, it is important to highlight that the Integrated Center for Diabetes and Hypertension of Ceará (CIHD), where this study was carried out, is highly concerned about the oral health of its patients. Dental care is provided at the CIHD and all patients with diabetes can have regular dental appointments for treatments and prevention.

The results of the DMFT index for both genders are in accordance with the latest regional figure reported by the Ministry of Health of Brazil in 2010, which indicated a mean of 27.2 teeth in people aged 65–74 years in the city of Fortaleza [23]. However, this value is slightly below the one found in a study conducted in the same city with people in the same age group– 30.7 teeth [24]. The periodontal conditions of the older people in the present study are better than those of people aged 65–74 years assessed in Project SB Brasil 2010 with regard to the presence of shallow (4 to 5 millimetres) and deep periodontal pockets (6 millimetres or more). The dental care provided at the Center for Diabetes and Hypertension of Ceará (CIHD) may explain the better oral condition of these patients compared to that of the general population of Northeastern Brazil.

Statistically significant difference (p<0.001) was found between genders with regard to the number of missing teeth in the DMFT index. The values found indicate that women experience tooth loss more than men. Among the studies referenced in this research, only the one by Colussi, Freitas and Calvo [25] presented results that are similar to the ones found in the present study.

Such findings may be explained by the fact that women seek dental care more often than men and are hence subjected to non-conservative and/or iatrogenic procedures throughout life [26]. On the other hand, the low rate of decayed teeth observed, much lower than the rate of filled teeth, may be due to the existing dental care provided by dentist-surgeons specially trained in the care of patients with diabetes.

Regarding the perception of xerostomia/dry mouth, 49.2% of the interviewees reported feeling moderate to severe dry mouth. With regard to gender, 41 (50%) women and 18 (47.4%) men reported moderate to severe xerostomia. A total of 27 (45%) individuals who used insulin and 32 (53.3%) individuals who did not use insulin reported moderate to severe xerostomia. This result is close to the results of the studies by Närhi et al. [27] and Collin *et al*. [28], in which over 50% of the interviewees complained of xerostomia. The perception of xerostomia may be explained by the use of medications with potential xerostomic effects– 92 (76.7%) patients used at least one drug with such side effects.

According to Coimbra *et al*. [29], most of times the causes of xerostomia are not directly related to the aging process or age-related diseases; they are, indeed, related to the prolonged use of certain medications like anticholinergics, tricyclic antidepressants and antihypertensives, especially diuretics.

The study by Lin *et al*. [30] suggests that patients diagnosed with type 2 diabetes who complain of xerostomia are more likely to suffer a decrease in salivary flow. Hyposalivation at different levels was observed in 111 (92.5%) patients, ranging from mild reduction to asialia. Because of the wide variety of tests for measuring salivary flow, many studies have presented conflicting results. The methodological heterogeneity and the lack of studies comparing salivary flow and xerostomia among diabetic groups–insulin-dependent and non-insulin dependent individuals–reveal the need for further studies on this issue. The association between low saliva production and moderate to severe xerostomia observed in this study was not significant (p = 0.489), showing that xerostomia is not associated with hyposalivation only.

With regard to stimulated salivary flow, only 13.2% of men and 4.9% of women presented normal values of salivary production. Normal salivary flow was found in 8.3% of insulin-dependent patients and in 6.7% of non-insulin dependent patients. Additionally, severe reduction of salivary flow was the most prevalent condition in both genders as well as in insulin-dependent and non-insulin dependent patients.

The change in salivary flow in diabetic patients is caused by multiple factors like the changes in the parenchyma of the salivary gland [31], glycosuria caused by mild hyperglycemia [32], and diabetes complications such as neuropathy, angiopathy and metabolic dyscontrol, decreasing the activity of the enzymes located in the salivary glands and hence affecting its function [33].

Older people who presented hyposalivation also presented a smaller number of teeth compared to a group of older people with normal salivation [10]. Older people with greater tooth loss and who use prostheses also suffer a decrease in salivary flow [34]. The failure in masticatory rehabilitation is a factor that can enhance hyposalivation.

It is known that the vast majority of older people present physiological salivary reduction, tooth loss, use prostheses, use long-term medications and are affected by chronic degenerative diseases. However, the 65-year-old diabetic patients assessed in the present research showed sialometric values lower than those found in studies with diabetic and non-diabetic individuals aged 60 [34, 35].

No statistically significant difference was found when comparing salivary flow between insulin-dependent and non-insulin-dependent older people. With regard to gender, stimulated salivary flow was higher in men (0.63±0.44ml/ min) compared women (0.47±0.41ml/min) (p = 0.05). This difference is contradictory to the results of the study by Lima *et al*. [36] and Cabrera *et al*. [37], in which there was no statistically significant difference in salivary flow by gender.

Since the research universe was focused on a single municipality, its results cannot be extrapolated and characterize a limitation of this study. However, the study was conducted in a large city of Brazil; therefore, its results may also be found in other places and hence allow the measurement of the impact of diseases in addition to serving as a basis for equity in public health care policies.

Thus, although the present study is limited to one single large reference hospital, it is expected to draw attention to the magnitude of the problem and the need for further research on the issue globally.

## Conclusions

Women in the present research presented significantly higher mean DMFT scores and tooth loss rates than men. A high prevalence of hyposalivation was observed in the current study, in which only 9 (7.5%) patients presented normal values of salivary production and 75 (62.5%) presented severe reduction.

No statistically significant association was found between the use of medications that caused xerostomia and salivation status. Reduced salivary flow was prevalent in the group assessed in the present research. However, this finding is not in accordance with the perception of xerostomia/dry mouth reported by the older patients. Such hyposalivation may be related to the many risk factors for reduction in salivary flow found in the group analyzed, particularly the advanced age, DM2 and tooth loss.

## References

[1] World Health Organization- WHO. Global Health data repository (GHO) data. http://apps.who.int/gho/data/node.main.688. 2012. (Accessed May 29, 2016).

[2] Carper MM, Traeger L, Gonzalez JS, Wexler DJ, Psaros C and Safren SA. The differential associations of depression and diabetes distress with quality of life domains in type 2 diabetes. *J Behav Med* 2014;37:501–510. doi: 10.1007/s10865-013-9505-x 23515932 10.1007/s10865-013-9505-x PMC3758402

[3] Peters SAE, Huxley RR and Woodward M. Diabetes as risk factor for incident coronary heart disease in women compared with men: a systematic review and meta-analysis of 64 cohorts including 858,507 individuals and 28,203 coronary events. *Diabetologia* 2014; 57:1542–1551. doi: 10.1007/s00125-014-3260-6 24859435 10.1007/s00125-014-3260-6

[4] Shi Y, Hu FB. The global implications of diabetes and cancer. *Lancet* 2014; 383: 1947–1948. doi: 10.1016/S0140-6736(14)60886-2 24910221 10.1016/S0140-6736(14)60886-2

[5] Oliveira JEP and Vencio S. *Diretrizes da Sociedade Brasileira de Diabetes*: *2013-2014/Sociedade Brasileira de Diabetes*; São Paulo: AC Farmacêutica, 2014.

[6] Ricci P, Blotière PO, Weill A, Simon D, Tuppin P, Ricordeau P, et al Diabètetraité: quellesévolutions entre 2000 et 2009 en France? *Bull Epidemiol Hebd* 2010; 42–43:425–431.

[7] Negrato CA and Tarzia O. Buccal alterations in diabetes mellitus. *Diabetol Metab Syndr* 2010; 2: 1–11. 20180965 10.1186/1758-5996-2-3 PMC2843640

[8] Takeuchi K, Furuta M, Takeshita T, Shibata Y, Shimazaki Y, Akifusa S, et al Risk factors for reduced salivary flow rate in a Japanese population: the Hisayama Study. *Biomed Res Int* 2015; 7:1–8 10.1155/2015/381821 PMC4332456 25705657

[9] Soares MSM, Passos IA, Maia RMF, Costa LJ and Veloso DJ. Fluxo salivar e consumo de medicamentos em diabéticos idosos. *Arq Odontol* 2004;40: 49–57.

[10] Samnieng P, Ueno M, Shinada K, Zaitsu T, Wright FA and Kawaguchi Y. Association of hyposalivation with oral function, nutrition and oral health in community-dwelling elderly Thai. *Community Dent Health* 2012; 29:117–23. 22482262

[11] World Health Organization—WHO. Oral health surveys: basic methods. 4Th ed Geneva: RH/EPID; 1997.

[12] Brokelman RBG, Daniel Haverkamp D, van Loon C, Hol A, van Kampen A, Veth R. The validation of the visual analogue scale for patient satisfaction after total hip arthroplasty. *Eur Orthop Traumatol* 2012; 3:101–105. doi: 10.1007/s12570-012-0100-3 22798966 10.1007/s12570-012-0100-3 PMC3389603

[13] Conceição MD. *Bom hálito e segurança*: *metas no tratamento da halitose*, 1rst ed. Campinas: Arte em livros, 2013; 105–112.

[14] Skaleric U, Schara R, Medvescek M, Hanlon A, Doherty F and Lessem J. Periodontal treatment by Arestinand its effects on glycemic control in type1 diabetes patients. *J Int Acad Periodontol* 2004; 6:160–5. 15536785

[15] Schara R, Medvescek M and Skaleric U. Periodontal diseaseand diabetes metaboliccontrol: a full-mouthdisinfection approach. *J Int Acad Periodontol* 2006; 8: 61–6. 16623181

[16] Koromantzos PA, Makrilakis K, Dereka X, Katsilambros N, Vrotsos IA and Madianos PN. A randomized, controlled trial on the effect of non-surgical periodontal therapy in patients with type 2 diabetes. Part I: effect on periodontal status and glycaemic control. *J Clin Periodontol* 2011; 38: 142–7. 10.1111/j.1600-051X.2010.01652.x 21114680

[17] Costa KL, Taboza ZA, Angelino GB, Silveira VR, Montenegro R Jr, Haas NA, et al The Influence of Periodontal Disease on Changes of Glycated Hemoglobin Levels in Type 2 Diabetics: a Retrospective Cohort Study. *J Periodontol* 2016, 26:1–13. 10.1902/jop.2016.160140 27562220

[18] Silva AM, Vargas AMD, Ferreira EF and Abreu MHNG. A integralidade da atenção em diabéticos com doença periodontal. *Cienc Saúde Colet* 2010; 15: 2197–206. 10.1590/s1413-81232010000400034 20694342

[19] Barros MBA, Francisco PMSB, Zanchetta LM and Cesar CLG. Tendências das desigualdades sociais e demográficas na prevalência de doenças crônicas no Brasil, PNAD: 2003–2008. *Ciênc Saúde Colet* 2011; 16:3755–68. 10.1590/s1413-81232011001000012 21987319

[20] Freitas LRS and Garcia LP. Evolution of prevalence of diabetes and associated hypertension in Brazil: analysis of national household sample survey, 1998, 2003 and 2008. *Epidemiol Serv Saúde* 2012; 21: 7–19.

[21] American Diabetes Association (ADA). Standards of Medical Care in Diabetes-2011. Position Statement/AmericanDiabetes Association. *Diabetes Care* 2011; 33:S11–61.

[22] Panarotto D, Rizzi AR, Tessari C, Brambatti KP, Atico MS and Severa A. Associação entre idade ao diagnóstico de diabetes do tipo 2 e o uso de insulina. *Revista da Amrigs* 2005; 49: 137–216.

[23] Ministério da Saúde. Projeto SB Brasil 2010: Pesquisa Nacional de Saúde Bucal–Resultados Principais. Brasilia (DF): Secretaria de Políticas de Saúde, 2011.

[24] Lima DLF, Cardoso Neto AM, Araújo KVC, Mendonça JS, Fernandes VO and Montenegro RM Junior. Comparison of oral status among diabetic and non-diabetic elders: a pilot study. *J Dent Rest* 2014; 1:016.

[25] Colussi CF, Freitas SFT and Calvo MCM. Perfil epidemiológico da cárie e do uso e necessidade de prótese na população idosa de Biguaçu, Santa Catarina. *Rev Bras Epidemiol* 2004; 7: 88–97.

[26] Guimarães KB, Meireles SS, Marques M, Sand Costa LJ. Condições periodontais em portadores de diabetes mellitus tipo 2 atendidos na Universidade Federal da Paraíba. *Rev Odonto Ciênc* 2007; 22:124–30.

[27] Narhi TO, Meurman JH, Ainamo A and Tilvis R. Oral health in the elderly with noninsulin-dependent diabetes mellitus. *Spec Care Dentist* 1996; 16:116–122. 9084324 10.1111/j.1754-4505.1996.tb00844.x

[28] Collin HL, Niskanen L, Uusitupa M, Töyry J, Collin P, Koivisto AM, et al Oral symptoms and signs in elderly patitents with type 2 diabetes mellitus. *Oral Surg Oral Med Oral Pathol Oral Radiol Endod* 2000; 90: 299–305. 10982950 10.1067/moe.2000.107536

[29] Coimbra F. Xerostomia. Etiologia e Tratamento. *R Port Estomatol Cir Maxilofac* 2009; 50:159–164.

[30] Lin CC, Sun SS, Kao A and Lee CC. Impaired salivary function in patients with noninsulin-dependent diabetes mellitus with xerostomia. *J Diabetes Complications* 2002; 16:176–9. 12039402 10.1016/s1056-8727(01)00174-x

[31] Murrah VA, Crosson JT and Sauk JJ. Parotid gland basement membrane variation in diabetes mellitus. *J Oral Pathol* 1985; 14: 236–46. 3921679 10.1111/j.1600-0714.1985.tb00487.x

[32] Malicka B, Kaczmarek U and Skośkiewicz-Malinowska K. Prevalence of xerostomia and the salivary flow rate in diabetic patients. *Adv Clin Exp Med* 2014; 23:225–33. 24913113 10.17219/acem/37067

[33] Carboni AMG, Carvalho LAC, Mello WR and Magalhães MHCG. Anomalias sistêmicas e bucais em pacientes com diabetes mellitus: revisão e caso clínico. *Diabetes Clínica* 2000; 4:62–68.

[34] Islas-Granillo H, Borges-Yañez SA, Medina-Solís CE, Galan-Vidal CA, Navarrete-Hernández JJ, Escoffié-Ramirez M, et al Salivary Parameters (Salivary Flow, pH and Buffering Capacity) in Stimulated Saliva of Mexican Elders 60 Years Old and Older. *West Indian Med J* 2014; 63:758–65. 25867562 10.7727/wimj.2014.036 PMC4668959

[35] Noboru Kuroiwa D, Ruiz Da Cunha Melo MA, Balducci I, Bortolin Lodi K, Ghislaine Oliveira Alves M and Dias Almeida J. Evaluation of salivary flow and drug interactions in patients with a diagnosis of diabetes mellitus. *Minerva Stomatol* 2014; 63:421–6. 25503343

[36] Lima AAS, Machado DFM, Santos AW and Grégio AMT. Avaliação sialométrica em indivíduos da terceira idade. *Rev Odonto Ciência* 2004;19:238–44.

[37] Cabrera MAS, Mesas AE, Rossato LA and Andrade SM. Fluxo salivar e uso de drogas psicoativas em idosos. Rev. Ass. Med. Bras 2007; 53: 178–181. 17568925 10.1590/s0104-42302007000200025

